# Supplementary material for: Double-Stranded RNA Binding Proteins in Serum Contribute to Systemic RNAi Across Phyla—Towards Finding the Missing Link in Achelata
Source: Int J Mol Sci. 2020 Sep 22;21(18):6967. doi: 10.3390/ijms21186967 (PMC7554946; doi:10.3390/ijms21186967)
Supplement: Supplementary file 1 [file ijms-21-06967-s001.zip › Supplementary materials gel shift paper.docx]

Supplementary materials

S1


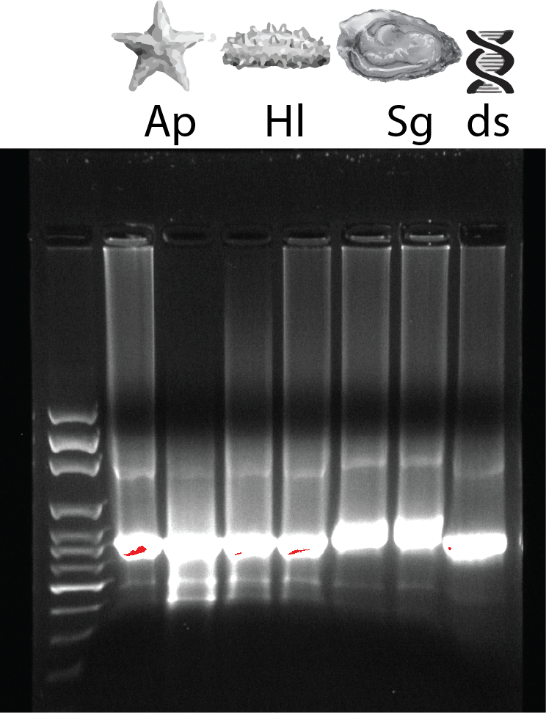


Re-run of *Acanthascter planci*, *Holothuria leucospilota*, and *Saccostrea glomerata* serum + dsRNA. A more noticeable gel shift was observed after running the samples for 90 minutes as opposed to 50 minutes.

S2

dsRNA sequences for eGPF and *Thenus orientalis* Insulin androgenic gland hormone

Sequence for *T. orientalis* IAG was retrieved from our 5^th^ walking leg transcriptome library.

The regions highlighted in yellow indicate the dsRNA sequence

Eukaryote eGFP:

ATGGTGAGCAAGGGCGAGGAGCTGTTCACCGGGGTGGTGCCCATCCTGGTCGAGCTGGACGGCGACGTAAACGGCCACAAGTTCAGCGTGTCCGGCGAGGGCGAGGGCGATGCCACCTACGGCAAGCTGACCCTGAAGTTCATCTGCACCACCGGCAAGCTGCCCGTGCCCTGGCCCACCCTCGTGACCACCCTGACCTACGGCGTGCAGTGCTTCAGCCGCTACCCCGACCACATGAAGCAGCACGACTTCTTCAAGTCCGCCATGCCCGAAGGCTACGTCCAGGAGCGCACCATCTTCTTCAAGGACGACGGCAACTACAAGACCCGCGCCGAGGTGAAGTTCGAGGGCGACACCCTGGTGAACCGCATCGAGCTGAAGGGCATCGACTTCAAGGAGGACGGCAACATCCTGGGGCACAAGCTGGAGTACAACTACAACAGCCACAACGTCTATATCATGGCCGACAAGCAGAAGAACGGCATCAAGGTGAACTTCAA

*T. orientalis*  IAG:

TGGGTATACATATATAGCCGTTGGGGTTCCTCAGGGGTGCAGGCGAAGCTCCAGCATTGGACCCAAACACACGCCCTTCGGCACTCCCTGCACCCGCCACCAGCACTCCTCTGAGCCCCATTTTTCCAAGCTTCTTCTCCAGCTTCGCAAGATCCTTCCCAAGAAACCTGCTCGACTTGCACCCTCCTCTCTGACTCTTTATCTTCTCCAAGGCTTCCCTTCCTCTCGAACTCTCCCCCGTCCTTAACTTTCCGAGTGAATACGGAGGAACCAAGCATTATCCTAGGCGTAAGTGTAAGACGCTTTCAGG

ATGTCACCACAAATCCTGCTCATTTTGGTGCTGATGGCGGGTATGCAGCCTCTCCACTCCATTTCGTACAACGTGTCCGGTGGGACGGTGGATTGTGGAGATATCGGAGCGACAATGAACGAAATTTGTCATAAGTTCTTGCCTTATTACAACGACGACTCCCATCAAAGGAAAAGGTCCGTCAGCACGAGCGTTAACGCCGACTCCAGCAGAAGGCAAGAGGCCCCAATCCAGCGGCATTACCACCCGAGGGCAACCCAGACCTTCCTGACAGACGCCGCCGCCAGCCCTCCTGACGCGGAAGGCACGCTGGCGAGGAGTAGGATCGCCTTCAAGCTGGTGAAGAACGAGGTGGCCTCGAGTCTGGTGAAGAGCCGTTTCCGCAGGGAAACTAACGTAAAGGACGAGTGCTGCAACGAAACGGAGCTCAGGCAGTGCTCCTCCAGGGAACTCCATGAATACTGTCAGGAGGTACCCGACGCCAGAGACGATTAA

CCAGAAATGAGGGGACGCGGGCTTTGAAGGAGGCTAGAATGCGAACGGCGATGGTTGTTGGGGGAAGAAATGTGAGACCTTCCTTCATGCTGATGAAAATTTAAAGCTTCTGGTGAAGACAGAGAGTCCAGCGTGTCATTGAAGTTTGTTGTGGGAAGAGAGTGAACATCAAGCCTTGTGAAAGGTGATAAGTCGATAGCGCATTGACGATAGTGAATGAAAACTGAGGAGGTTCAAGTGACAGGGGAAGATAATGAATGTTATGTCTTTTAAGGAGATAATGCTAGTGAAGTGTAAGGGGATGATGGTGAAAATTCATTATCATCTTTTTTTTACTTTATGGAAGAGAGTAAAGGTCGAACTAACAGTGAAAAGAGAAAATGACCACCAGTTCCCCTTTCTCATTATTTTATATACCGTCTTGTATCATCTTGCTACCTTGTTGATTAGACTCATTCTACACACACAAAAATATGTGTACTTTGATACACGCATACACTGACGAGCTGGGATAAATATGCAAAC

ACGCATACTTACAGAAATATTTCACAGACACACGCAAATGCAGTCACGAACGCACACATATGAAGAGTACAACTTGAAGGTTATTAGAAATGTACTTCTGTCATCATGAAACTTATGTCAAGCAATCAAAGGTACTTATCTTTCCCTTGTACTATGAACAACTTACGAATATATCCATACTGTTTCTTACATTAGTAAAATTGCATTACAAAAAAAAAAAAAAAAAAAAAAAAAAAAAAAAAAAAAAAAAAAAAAAAAAAAAAAAAAAAAAAAAAAAAAAAAAAACAAAAAAAAAAAAATAAAGA
